# A Mixed Methods Framework for Psychoanalytic Group Therapy: From Qualitative Records to a Quantitative Approach Using T-Pattern, Lag Sequential, and Polar Coordinate Analyses

**DOI:** 10.3389/fpsyg.2020.01922

**Published:** 2020-08-11

**Authors:** Eulàlia Arias-Pujol, M. Teresa Anguera

**Affiliations:** ^1^FPCEE Blanquerna, Ramon Llull University, Barcelona, Spain; ^2^Faculty of Psychology, Institute of Neurosciences, University of Barcelona, Barcelona, Spain

**Keywords:** mixed methods, QUAL-QUAN-QUAL integration, group therapy, adolescents, psychotherapist interactions

## Abstract

Conducted within a mixed methods framework, this study focuses on the conversation-facilitation role of a lead therapist during group psychotherapy with adolescents. Conversation is an essential component of psychoanalytic psychotherapies and there is growing interest in describing and studying the impact of conversational techniques. One way to do this is to report on specific approaches, such as questioning, paraphrasing, and mentalization in intervention turns and to analyze their impact on the therapist-patient relationship. The main aim of this study was to investigate differences in communication strategies used by a lead therapist in the early and late stages of therapy with six adolescents aged 13–15 years. We employed a mixed methods design based on systematic direct observation supplemented by indirect observation. The observational methodology design was nomothetic, follow-up, and multidimensional. The choice of methodology is justified by our use of an *ad hoc* observation instrument for communication strategies combining a field format and a category system. We analyzed interobserver agreement quantitatively by Cohen’s kappa using GSEQ5 software. Following confirmation of the reliability of the data, we analyzed the lead therapist’s conversation-facilitation techniques in sessions 5 and 29 of a 30-session program by quantitatively analyzing what were initially qualitative data using T-pattern detection (THEME v.6 Edu software), lag sequential analysis (GSEQ5 software), and polar coordinate analysis (HOISAN v. 1.6.3.3.6. software and R software). The results show changes in the techniques used from the start to the end of therapy. Of the 28 communication strategies analyzed, three were particularly common: questioning and paraphrasing in session 5 and questioning and mentalization in session 29. This mixed methods study shows that combined use of T-pattern detection, lag sequential analysis, and polar coordinate analysis can offer meaningful and objective insights into group psychotherapy through the lens of the therapist.

## Introduction

The use of mixed methods in psychotherapy research has grown in recent years (Bartholomew and Lockard, 2018; Del Giacco et al., 2019, 2020; Halfon et al., 2019; Roberts and Allen, 2019; Venturella et al., 2019). Researchers working as psychotherapists have underlined the need to integrate the objectivity of quantitative methods with the creativity of qualitative and mixed methods in order “to put the flesh of clinical meaning on the bones of quantitative outcomes” (Target, 2018, p. 18). Psychotherapy research covers a field of great complexity. Some of this flexibility can be captured and understood through the analysis of qualitative and quantitative data within a mixed methods design to shed light on what lies beneath multimodal interactions that precede change in psychotherapy. A mixed methods design offers an objective and scientifically rigorous yet flexible approach for capturing change and continuity over the course of psychotherapy. An observational methodology is perfectly suited to the study of spontaneous behavior in natural settings (Anguera et al., 2018), and as such, is ideal for analyzing the regular interactions that occur between therapists and patients in a range of psychotherapy settings (individual, group, or family therapy) and, more specifically, in psychoanalytic therapy settings (Arias-Pujol and Anguera, in press). The process for analyzing change in psychotherapy is well established and plenty of opportunities exist within this process (from the definition of the research question to the interpretation of systematically collected and recorded data) to integrate both qualitative and quantitative elements.

Conventionally speaking, mixed methods studies integrate qualitative and quantitative perspectives (Johnson et al., 2007; Creswell and Plano Clark, 2017; Onwuegbuzie et al., 2018) in drawing on all types of data, including observational datasets, narratives, in-depth interviews, survey results, and measures from physiological and other tests, including repeated measures taken over the course of a single study. The means by which these data can be linked has grown exponentially, giving rise to numerous conceptual nuances, a long list of study designs, and a future that promises to end decades of methodological confrontation.

The very essence of the observational methodology consists of seeking complementarity through the integration of qualitative and quantitative elements. A key strength of the observational methodology is that it rigorously guarantees quality through the objective analysis of rigorously collected and processed qualitative data that can be analyzed robustly and quantitatively without loss of information richness (Anguera et al., in press).

Observational methodology is thus itself a mixed methods approach (Anguera et al., 2017a). Although relatively novel, it has shown enormous promise and is growing in popularity in a range of scientific fields, including psychology. In their review of mixed methods in psychotherapy research, Bartholomew and Lockard (2018) reported that a considerable proportion (32.26%) of these studies have focused on group interventions. While most studies have been conducted in adults, some have been conducted in adolescents (Down et al., 2011) and children with emotional and behavioral problems in groups (Swank and Shin, 2015) and individual psychodynamic play therapy (Halfon et al., 2016). Our group has used observational methodology to analyze group psychotherapy in previous studies (Vaimberg, 2010; Roustan et al., 2013; Arias-Pujol and Anguera, 2017; Alcover et al., 2019) and in the empirical part of this article. In the latter we demonstrate the different steps involved in the QUAL-QUAN-QUAL transformation of data and show how the “connecting” method (Creswell and Plano Clark, 2017) is an ideal way to link qualitative and quantitative elements within a systematic observation framework.

There is growing interest in describing and studying the impact of psychoanalytic therapy techniques (Midgley et al., 2018) from different perspectives, with researchers continuing to search for answers to the question “what works for whom?” (Fonagy et al., 2015). Different approaches to responding to this question have been adopted: some studies have taken a microanalytical approach based on the psychophysiological responses of therapists and patients (Steffen et al., 2014; Kleinbub, 2017), while others have analyzed the impact of therapist empathy and challenge on psychophysiological responses from patients (Voutilainen et al., 2018).

From a mixed methods perspective this question can be addressed by analyzing communication and therapeutic discourse interactions using an *ad hoc* observation instrument (Arias-Pujol et al., 2015; Arias-Pujol and Anguera, 2017; Del Giacco et al., 2019, 2020). Conversation and the therapeutic alliance are essential components of psychoanalytic therapy (Manzano et al., 2018). In the case of adolescents, creating a therapeutic alliance is crucial to preventing therapy dropout (O’Keefe et al., 2018). As the therapy unfolds, an alliance is formed between the therapist and participants (Tanzilli et al., 2018) that allows the therapist to communicate increasingly complex content concerning the here-and-now of the sessions. In the early stages of therapy, this intrapsychic content may not be understood by the patient, and might even lead to resistance, especially in adolescents (Oetzel and Scherer, 2003; Lavik et al., 2017, 2018); it can only emerge once a climate of trust has been created (Sagen et al., 2013). Interventions of this type are aimed at increasing the patient’s capacity for mentalization, which is a process by which people make sense of themselves and each other (Fonagy, 1991). In group sessions, therapist interventions designed to build capacity for mentalization show that behaviors are motivated by emotions, thoughts, fantasies, and wishes. Although it is recognized that everybody has their own mind, the group participants come to see the benefits of sharing points of view and empathizing with other people’s experiences (Torras de Beà, 2013). A previous study by our group (Arias-Pujol and Anguera, 2017) that analyzed conversation turn-taking in adolescent group therapy showed that four main roles were played by the lead therapist: (1) she did not facilitate interventions by all group members uniformly, (2) she encouraged turn-taking from more inhibited participants, (3) she facilitated conversation from the early stages of therapy, and (4) she promoted the capacity to mentalize toward the end of therapy.

The aim of this new study, conducted within a mixed methods framework, was to investigate potential differences in the communication strategies used by a lead therapist in earlier and later stages of therapy. The specific aim was to use T-pattern detection, lag sequential analysis, and polar coordinate analysis to detect changes in the communication flow between a lead therapist and her patients (in this case, adolescents), analyzing the specific techniques used and their impact on the therapist-patient relationship.

## Materials and Methods

### Design

The specific design was nomothetic/follow-up/multidimensional (N/F/M) (Anguera et al., 2001; Sánchez-Algarra and Anguera, 2013). It was nomothetic because we observed different participants (lead therapist, co-therapist, and group members), follow-up because we analyzed two sessions and their content (one session each from the beginning and the end of the therapy), and multidimensional because we analyzed 15 communication strategy dimensions using an *ad hoc* observation instrument (Arias-Pujol and Anguera, 2017). Both direct observation (Sánchez-Algarra and Anguera, 2013) and indirect observation (Anguera et al., 2018) techniques were used. The observation was participative, given that the psychotherapist interacted with the adolescents. The recommendations of the *Guidelines for Reporting Evaluations based on Observational Methodology* (GREOM) (Portell et al., 2015) and the *Methodological Quality Checklist for Studies based on Observational Methodology* (MQCOM) (Chacón-Moscoso et al., 2019) were followed.

### Participants

The group therapy sessions were conducted in the Eulàlia Torras de Beà Foundation (FETB) Center for Child and Adolescent Mental Health (Barcelona, Spain) with six adolescents (four boys and two girls) aged 13–15 years, an expert lead therapist, and a co-therapist. All the adolescents had difficulties with learning and interpersonal relationships.

This research forms part of a broader project involving an 8-month intervention developed to enhance the mentalization and communication capacities of adolescents, whose parents attended parallel sessions on parenting. The goal of the therapists was to facilitate interaction among all the group members by creating an atmosphere of emotional security and support (Torras de Beà, 2013).

Written informed consent was obtained from the parents of the minors in accordance with the principles of the Declaration of Helsinki and the Ethical Code of the General Council of the Spanish Official College of Psychologists. Approval by an ethics committee was not required as per applicable institutional and national guidelines and regulations. The participants were informed that they were being filmed and agreed accordingly, and were shown the location of the video cameras, positioned discretely to minimize reactivity bias. They were guaranteed that their identity and privacy would be protected at all times. For this, pseudonyms were used in the transcripts and encodings of the material. The study was approved by the head of the Eulàlia Torras de Beà Foundation (FETB) Research Department. Regulatory provisions regarding clinical research in humans of the European Union (Good Clinical Practice for Trials on Medicinal Products in the European Community: EEC 111/3976/88-EN) and of Spain (Royal Decree 561/1993) were applied.

### Instruments

#### Observation Instrument

The observation instrument, developed *ad hoc* for a previous study, combined field format and several category systems (Arias-Pujol and Anguera, 2017). The category systems were built on 15 dimensions proposed for analysis of communication strategies. A category system was built from each dimension (except for some single-category dimensions) that fulfilled the requirements of exhaustiveness and mutual exclusivity. The ‘turn’ dimension was observed directly, while the other 14 dimensions were observed indirectly. A total of 28 categories resulted for the dimensions (Table 1).

**TABLE 1:** Dimensions and category systems in the observation instrument for therapists and patients (adapted from Arias-Pujol and Anguera (2017).

Dimension and category systems	Description
Dimension DYN **Facilitating conversation** Categories: FF, FO, RP, RT, QA, QC, and QV	**Facilitating conversation**. Suitable questions or requests to start or enhance dialogue; routines such as greetings and other conversational rituals; requests for clarification; verification questions; full or partial repetitions of a previous intervention in the form of a statement or a question; vocalizations indicating that the communication channel is still open.**FF** = Phatic function. Vocalization indicating that the communication channel is still open. It indicates continued attention and cooperation, without the addition of new information. Typical vocalizations are “hmmn,” “hum,” or “aha.”**FO** = Conversational routines or rituals, such as greetings or expressions of gratitude.**RP** = Paraphrasing. Total or partial reproduction of a previous utterance in the form of a statement not a question. This could be an answer to a request for clarification or it could have a phatic function, such as, for example, when the speaker simply echoes what a person has just said, indirectly encouraging them to continue.**RT** = Bringing back a topic of conversation. Intervention in which a participant brings back a subject previously brought up by another participant after a change of subject (CT) or interruption, thus making sure it is not forgotten.**QA** = Questioning. Request, expressive question, or series of adequate questions to start or promote dialogue and keep the main topic of conversation flowing. The person gives the turn to another person and shows interest in them.**QC** = Clarifying question. Question asking for clarification about what is happening. The person intervenes to clarify their own confusion and/or surprise in the form of a question. The speaker asks about a particular topic, doubt, or puzzlement, or about expressions, gestures, noises, or laughter he/she has not understood. It is a strategy used by the therapist when the adolescents are “doing their own thing.”**QV** = Repetition of a previous statement in the form of a question. It is used to confirm what has just been said. It has a phatic function, as the speaker is conveying that the communication channel is still open. It can also be a strategy to emphasize a particular word or intervention.
Dimension**Mentalization**:MNT	**MentalizationMNT** = Interventions focused on promoting thought, reflection, and understanding of oneself and one’s relationships with others. They seek to stimulate the ability to understand what is happening in the minds of others. They are used by the therapist and can be directed at an individual or at the group as a whole. They include emphatic interventions, which put words to other participants’ feelings.
Dimension**Expressivity**Categories: RA, EC, CD, and RB	**Expressivity.** Interventions and answers manifesting the thoughts and/or feelings of the person speaking, the conversation flows.**RA** = Interventions that answer a question.**RP** = short answer: yes or no.**EC** = sequences of words that continue the main subject of conversation; it is not an answer.**CD** = Interventions that give a new approach to the same subject.
Dimension**Defensive**categories: RD_N_P and CT	**Defensive expressionsRD_N_P** = Interventions in which the participant avoids answering a previous question; verbalizations expressing the opposite of what has been said or done; projection of conflicts onto others.**CT** = changing subject.
Dimension**Dislike**categories: ED and PD	**DislikeED** = Interventions expressing dislike, disagreement, or distaste.**PD** = Interventions expressing defiance.
Dimension**Ordering**:ORD	**Ordering.ORD** = Prescriptive verbalizations, authoritarian demands (including exclamations).
Dimension**Humor**categories: R and EO	**HumorEO** = Interventions with a clearly ironic/wry intention, jokes, jibes.**R** = laughter.
Dimension**Confrontation**:CFR	**ConfrontationCFR** = Verbal interventions used by participants to express what they feel is happening in the group or see in some of their peers. They mirror the behavior of another.
Dimension**Exclamation**:EX	**ExclamationEX** = Onomatopoeic word or words indicating a strong emotion of surprise, joy, or sadness.
Dimension**Degradation of vocal behavior**:S4	**Degradation of vocal behaviorS4** = Failed spontaneous interventions, interventions that progressively become weaker, abandoning turn.
Dimension**Whispering**:S5	**WhisperingS5** = Talking in a low voice, with the intention of being heard by only a few people, establishing complicity. It leads to confused murmuring.
Dimension**Touching**:TO	**TouchingTO** = Intentional physical contact with another person.
Dimension**Noise**categories: MO, S2, and S3	**Noise.** Noise or noises produced:**S2** = by a person, through their body (e.g., sneezing, burping, and clapping)**S3** = interaction with an object (e.g., chair, table, and wall).**MO** = movement.
Dimension**Surrounding noise:**S1	**Surrounding noiseS** = Sounds from outside the therapy room that are loud enough to be clearly heard.
Dimension**Silence**:Q	**Silence.Q** = No words. Indicates no behavior.
Dimension**Turn:**Turn	**T =** Lead therapist.**coT =** Co-therapist.**G =** Gabriel (pseudonym).**D =** Danny (pseudonym).**JM =** John M. (pseudonym).**F =** Fred (pseudonym).**L =** Lucy (pseudonym).**M =** Megan (pseudonym).*Pseudonyms have been used to protect confidentiality.*

#### Recording and Analysis Instruments

The recording instrument used was the freeware GSEQ5, v.5.2^1^ (Bakeman and Quera, 1996, 2011), which allowed the sessions to be coded in accordance with the observation instrument. The obtained data were type II data (Bakeman, 1978), and, therefore, concurrent and event-based. GSEQ5 was also used to calculate agreement.

Regarding the analyses, T-patterns were detected using the freeware THEME v.6 Edu^2^ (Magnusson, 1996, 2000, 2020), lag sequential analysis was performed using GSEQ5, polar coordinates were analyzed using the freeware^3^ HOISAN v. 1.6.3.3.6. (Hernández-Mendo et al., 2012), and vectors were graphed using R (Rodríguez-Medina et al., 2019).

### Procedure

This research was part of a group psychotherapy program consisting of 30 sessions, 24 of which were transcribed to capture conversation turn-taking. To delimit the observation unit, we used interlocutor and syntactic criteria in a complementary manner (Anguera, in press; Krippendorff, 2013). As mentioned, the data were type II data (Bakeman, 1978), which materialize code matrices as obtained in the quantitizing process; these qualitative data from the recording (see vignettes in Tables 2, 3) were systematized through observation-instrument coding and computerized recording. The code matrices contain rows (a separate row for each observation unit) that show the codes for co-occurrences of simultaneous behaviors for the different dimensions of the observation instrument. Quantitizing is crucial in the mixed methods framework (Anguera et al., 2017a; Anguera, 2020; Anguera et al., in press), as it enables access to the second QUAL-QUAN-QUAL phase; the fact that the code matrices are quantitatively analyzed allows for the crucial step that connects functions and that permits the integration of qualitative and quantitative elements (Plano Clark and Sanders, 2015), in such a way that initially qualitative data can be analyzed quantitatively (Anguera et al., 2018).

**TABLE 2:** Sample clinical vignette for the initial session.

Although this session was session 5 in the group psychotherapy program, it was the first one held with all the participants.
The lead therapist (T) plays a very active role, encouraging participation so that the adolescents can get to know each other. She asks them about their names, ages, hobbies, how they get to the therapy sessions, what expectations they have about the group, what they like, and what annoys them. They contribute by talking about their experiences with teachers, classmates, and out-of-school activities.
Example:
T- What about you, Megan? (QA)
M- Well, the girls in my class, they go together to a corner (of the shopping center)… (RA)
T- To a corner (RP)
Laughter (R)
M- A corner… (EC)
T-Hmm (FF)
M- And they start smoking, they smoke, drink… (EC)
T- They smoke and drink (RP)
M- Some of them, yes, they do… (EC)
G- Well, then, I’m going there too! (EC)
Laughter (R).

**TABLE 3:** Sample clinical vignette for the final session.

Everyone is present in this session and they chat as they enter the office from the waiting room. The dialogue is fluid with a lot of joking and laughing. The participants talk about the end of the therapy and the school year. The lead therapist (T) wants to know their opinions about the experience and highlights the changes that have occurred. Many conversations are interrupted by jokes and changes of subject. T tolerates this, comments that they have got to know each other, and that it is now hard to say goodbye.
Example:
G notices L’s shoes and they start talking about the size of their shoes and compare them with the T’s shoes. They then look at the size of their hands.
T- You are noticing your changes, the changes in others, and in the end this is how you see yourselves, how the others see you, whether you like yourselves or not… (MNT)
L- If we have liked ourselves here? (QA)
G- Yes (RB)
T- Also here… I imagine that everyone is thinking: what do they think of me, how do they see me, what image of me am I giving? (MNT)
G- Ugly! (EO)
They all laugh (R)
D (talking to G) – Bad imitation of your father, your grandfather… (CFR)
G (going on with the joke) – great-grandfather… (EO)
M (talking to F who is chewing on a part of his sweater) – Hey, sweater taste good? (EE QA)
Amidst jokes, touching, and laughter, they then start to talk about things people do when they are nervous. L bites her nails, G chews on a pen, D can’t stop moving his legs…
T- These are things that you say to each other, that you see in yourselves and in others. (MNT).

The reliability of the data was confirmed by calculating Cohen (1960, 1968); the obtained values of between 0.897 and 0.939, according to Landis and Koch(1977, p. 165), can be interpreted as “almost perfect agreement”.

#### Data Analysis

For the current study, we compared the content of two sessions in order to showcase an innovative methodological development in group psychotherapy in which qualitative records from the two sessions underwent a powerful quantitative analysis within a mixed methods framework.

The first session was an early session (session 5, the first with the full group), while the second one was a session from the end of therapy (session 29, held 7 months later, just before the farewell/end-of-treatment session). Once the data had been validated and transformed into code matrices, sessions 5 and 29 were analyzed in depth using three scientifically grounded and specific categorical data techniques: T-pattern detection, lag sequential analysis, and polar coordinate analysis (with the therapist as the focal subject). These quantitative techniques are highly appropriate for the analysis of qualitative data collected by direct observation (Anguera et al., 2017b) and indirect observation (Anguera et al., 2018) and suitably organized in code matrices within the framework of a mixed methods study (Anguera et al., in press). To date, the three techniques have been applied in combination in the fields of education (Santoyo et al., 2017; Escolano-Pérez et al., 2019) and sport (Tarragó et al., 2017).

##### T-pattern detection

T-pattern detection was proposed and developed by Magnusson (1996, 2000, 2005, 2016, 2018, 2020). T-patterns, or temporal patterns, are essentially a combination of events that occur in the same order, separated by temporal distances that remain invariant over time. The basic premise of T-pattern detection is that the interactive flow or chain of behaviors consists of structures of variable stability that can be visualized through the detection of underlying T-patterns (Suárez et al., 2018; Portell et al., 2019; Santoyo et al., 2020). As indicated by Magnusson(2020, p. 2): “As a Mixed Methods approach, T-pattern analysis […] passes repeatedly between qualitative and quantitative analyses, from data collection logging the occurrences of qualities (categories) and their real-time (quantitative) locations resulting in time-stamped data, here T-data, to the detection of T-patterns (qualities) […], typically followed by both qualitative and quantitative analyses of the detected patterns.” T-pattern analysis involves the use of an algorithm that calculates temporal distances between codes of behaviors, analyzing the extent to which the critical interval remains invariant relative to the null hypothesis. It requires the use of systematized data (usually in the form of code matrices) for which the duration of each co-occurrence has been recorded (Anguera et al., 2018). As indicated by Magnusson (1996, 2000, 2020), a T-pattern, Q, consists of *m* ordered components, X_1__…__*m*_, that are recurrent, where each temporal co-occurrence of behaviors (called event-types) is a T-data. A T-pattern can be characterized as follows, considering variations in distances between consecutive behaviors (Magnusson, 2020):

*Q* = *X*_1_ [*d*_1_,*d*_2_]_1_ *X*_2_ [*d*_1_,*d*_2_]_2_. *X*_*i*_ [*d*_1_,*d*_2_]_*i*_ *X*_*i*__+__1._ *X*_*m*__–__1_ [*d*_1_,*d*_2_] _(m__–__1__)_ *X*_*m*_,

where *X* is an event-type or a T-pattern. The general term *X*_*i*_ [*d*_1_, *d*_2_]*i X*_*i*__+__1_ means that, within occurrences of the pattern, after *X*_*i*_ occurring at *t* statistically significantly more often than expected by chance, *X*_*i*__+__1_ occurs within interval [*t* + *d*_1_, *t* + *d*_2_], or short [*d*_1_, *d*_2_], called a critical interval (Anguera et al., in press).

Microanalyses are also possible and very useful (Anguera, 2005). These analyses are run in THEME v. 6 Edu, which offers different settings that can be modified to obtain complementary results. Combined analysis of these results can provide a better understanding of interactive transitions over time. THEME provides all the necessary features to analyze the data and presents the results graphically as dendrograms or tree diagrams.

Two parameters necessary for each analysis are the minimum number of occurrences and the level of significance. We set the minimum number of occurrences to 30 and the significance level to *p* < 0.005. Note that the method applied in this research was rather unconventional, as the temporal distance parameter was set to 1 in all cases. This method was chosen because of the nature of the data (type II).

While T-pattern detection has been used in a wide range of fields, including clinical psychology (Blanchet et al., 2005; Haynal-Reymond et al., 2005; Merten and Schwab, 2005; Plumet and Tardif, 2005; Horn and Magnusson, 2016; Woods et al., 2016), its application to group therapy with adolescents is novel.

##### Lag sequential analysis

This technique, proposed by Bakeman (1978), aims to detect the existence of patterns of behavior within categorical data corresponding to regular behaviors that are not due to random effects. Lag sequential analysis one or more given behaviors (any that, by hypothesis, are assumed to generate or initialize a behavior pattern), one or more conditional behaviors (for which we wish to test the existence of a statistical association with a given behavior), and lags (positive, negative, or both). Behaviors with positive and negative lags occur after and before the given behavior, respectively. The number of the lag indicates the order in which it occurs.

Lag sequential analysis can operate with five types of data: event sequence data, state sequence data, timed state sequence data, interval sequence data, and multi-event sequence data. The first four were designated by Bakeman (1978) and were later slightly modified by Bakeman and Quera (1996, 2011) when building the SDIS-GSEQ software (precursor of the current GSEQ5). A minimum of 30 data items (30 code matrix rows) is required for the results to be valid (Bakeman and Gottman, 1987). Since lag sequential analysis works with code matrices (Anguera et al., 2018), it can be used to detect regularities (patterns of behavior) that show the structure of interactive episodes (Bakeman, 1978, 1991; Bakeman and Gottman, 1987; Bakeman and Quera, 1996, 2011; Quera, 2018); this is very useful in clinical psychology, especially when we want to detect regularities at different points in time.

Once the conditional behaviors and lags of interest have been defined, as per Bakeman (1978), a matching frequencies table based on the gicen behavior is generated and this is then used to generate a probability table showing expected and conditional probabilities. Expected probabilities indicate the extent of random effects, while conditional probabilities provide the residual values that indicate whether or not the relationship with the given behavior is significant that at each lag. It is recommended to apply the adjustment proposed by Allison and Liker (1982), incorporated in SDIS-GSEQ, as it expresses the results as adjusted residuals.

Once the adjusted residuals have been obtained, the pattern (or patterns) of behavior is (are) “constructed,” starting with the proposed criterion behavior in each case. Each lag (whether positive or negative) will include the conditional behavior(s) with a significant adjusted residual value: >1.96 when the relationship is activation and <−1.96 when the relationship is inhibition (for a significance level of *p* < 0.05).

So that researchers can consider where the structure conventionally ends, i.e., to end the interpretation purposes of the obtained structure, interpretation guidelines should be applied (a) when there are no more lags with statistically significant behaviors, (b) when there are two consecutive empty lags, or (c) when there are several statistically significant behaviors in two consecutive lags and the first of the lags is considered the MAX LAG (Anguera et al., in press; Sackett, 1979).

Lag sequential analysis can be applied, in both direct and indirect observation, to a complete session, part of a session, parts of different sessions (e.g., the first few minutes of a series of sessions), or series of complete sessions. It therefore offers enormous flexibility in addressing different research questions. It requires data for which the sequence of occurrence of concurrent behaviors has been recorded and it can be run in any of the following programs: GSEQ5 v. 5.2 (Bakeman and Quera, 2011) and GSEQ5 (Bakeman and Quera, 2011), which allow various simultaneous criterion behaviors, or HOISAN v. 1.6.3.3 (Hernández-Mendo et al., 2012), which only allows one criterion behavior.

Lag sequential analysis has been successfully applied in many direct and indirect observation studies conducted over the past 25 years in clinical psychology (e.g., Martínez del Pozo, 1993; Arias-Pujol and Anguera, 2004, Arias-Pujol and Anguera, 2005; Roustan et al., 2013; Arias-Pujol et al., 2015; Venturella et al., 2019; Del Giacco et al., 2020).

##### Polar coordinate analysis

Polar coordinate analysis, an analytical technique proposed by Sackett (1980), is based on building a map that shows the statistical association between different behavior codes, and specifically between a behavior that is considered central or core, called the focal behavior, and all other behaviors, called conditional behaviors. The goal is to determine if there is a relationship, and if there is one, its type and intensity. This technique, which considers as data the adjusted residuals obtained in the lag sequential analysis, complements the prospective (forward feeding) and retrospective (backward feeding) perspectives, allowing us to observe how the relationship between focal behavior and conditional behaviors evolves over time. This analysis is therefore based on prospective and retrospective perspectives. Sackett (1980) applied Bakeman’s (1978) concept of prospectivity, but considered retrospective lags feeding forward from negative lags, going from a lag of −5 to a lag of −4, from −4 to −3, and so on successively, in an approach open to criticism. Anguera (1997) proposed a promising new concept, called genuine retrospectivity – included in the analysis algorithm on programming the HOISAN software – that allows backward feeding, from lag 0 to −1, from lag −1 to −2, and so on (Gorospe and Anguera, 2000; Gorospe et al., 2005).

Sackett (1980) ingeniously used the *Z*_*sum*_ statistic proposed by Cochran (1954), providing a powerful means of data reduction provided the data are independent. He applied it to the obtained adjusted residual values (which are independent of each other because they each respond to a different calculation given that the lags are different) considering the criterion behavior of the sequential analysis as the focal behavior and the conditional behaviors in positive lags to obtain the prospective *Z*_*sum*_ values. He applied the same method (but using conditional behaviors in negative rather than positive lags) to obtain the retrospective *Z*_*sum*_ values. Note that the number of positive and negative lags must be the same (Sackett, 1980). Experience to date (Sackett, 1979; Anguera and Losada, 1999) indicates that at least five prospective (e.g., lags +1 to +5) and five retrospective lags (e.g., −1 to −5) to be analyzed (Anguera et al., 2018).

From the prospective and retrospective *Z*_*sum*_ values, Sackett (1980) proposed a vectorialization of the relationships between focal behavior and conditional behaviors. Each vector has length or radius $L\,e\,n\,g\,t\,h=\sqrt{{\left(Z_{s\,u\,m\,\mathrm{prospective}}\right)}^{2}+{\left(Z_{s\,u\,m\,\mathrm{retrospective}}\right)}^{2}}$ and an angle ϕ = *Arcsen*$\frac{Z_{s\,u\,m\,\mathrm{retrospective}}}{\text{Length}}$.

As many vectors as conditional behaviors are obtained, all graphically with their origins in the focal behavior. Because the prospective and retrospective *Z*_*sum*_ values have a positive or negative sign, the corresponding vectors can be plotted such that the prospective and retrospective values will be displayed along the horizontal (*X*)-axis and the vertical (*Y*)-axis, respectively.

The meaning of the vectors varies in function of the quadrant in which they are located, and the position of a vector in one quadrant or another is determined by the combination of positive or negative signs on the prospective and retrospective *Z*_*sum*_ values:

Quadrant I (+ +): the focal and conditional behaviors activate each other.

Quadrant II (− +): the focal behavior inhibits and is activated by the conditional behavior.

Quadrant III (−−): the focal and conditional behaviors inhibit each other.

Quadrant IV (+ −): The focal behavior activates and is inhibited by the conditional behavior. Vector length indicates the strength (statistical significance) of the association between the focal and conditional behaviors.

Like T-pattern detection, polar coordinate analysis has been used in a wide range of fields, including clinical psychology (Arias-Pujol and Anguera, 2017; Rodríguez-Medina et al., 2018; Alcover et al., 2019; Del Giacco et al., 2020).

## Results

The way in which the therapist and the adolescents communicated with each other changed from session 5 to 29 and the qualitative changes detected were confirmed quantitatively within a rigorous analytical framework. Sample clinical vignettes for each session are reproduced below.

Tables 4A,B shows the records corresponding to the vignettes in Tables 2, 3 for multi-event sequence data and according to the syntax of the GSEQ5 program. These data make up an .SDS file, compiled for the program to check for formal errors and generating an .MDS file once verified as correct.

**TABLE 4:** Fragment of record with multi-event sequence data using the syntaxis of the GSEQ5 program.

(A) Session 5	(B) Session 29
Multi-event	Multi-event
($HUM = R EO)	($HUM = R EO)
($DIS = ED PD)	($DIS = ED PD)
($NOI = MO S1 S2 S3)	($NOI = MO S1 S2 S3)
($TO = TO)	($TO = TO)
($EX = EE)	($EX = EE)
($WHI = S5)	($WHI = S5)
($ORD = DO)	($ORD = DO)
($DIN = QA QACL FF FO RP PV RT)	($DIN = QA QACL FF FO RP PV RT)
($MNT = MNT)	($MNT = MNT)
($CFR = CFR)	($CFR = CFR)
($EXP = RA EC CD RB)	($EXP = RA EC CD RB)
($DEF = RD_N_P CT)	($DEF = RD_N_P CT)
($Q = Q)	($Q = Q)
($TURN = O G D JM F L M);	($TURN = O G D JM F L M);
T QA.	T MNT.
M RA.	L QA.
T RP.	G RB.
R.	T MNT.
M EC.	G EO.
T FF.	R.
M EC.	D CFR.
T RP.	G EO.
M EC.	M QA.
G EC.	T MNT/
R/	

****(A)****Corresponding to session 5 (vignette from Table 2), and****(B)****corresponding to session 29 (vignette from Table 3). The first part contains, according to the syntax of the GSEQ program, the data type and the code declarations corresponding to the observation instrument*.*

The results of the three techniques (T-pattern detection, lag sequential analysis, and polar coordinate analysis) are presented below.

### T-Pattern Detection

For both sessions, the records obtained were transformed using the GSEQ5 program to adapt them to the syntax of the THEME program, which requires two files: the VVT.VVT file corresponding to the observation instrument, and the .RDT file corresponding to the recorded data. Tables 5A–C shows the VVT.VVT file and the respective records corresponding to the vignettes in Tables 2, 3, maintaining a conventional and constant distance according to the THEME syntax.

**TABLE 5:** Fragment of record using the syntaxis of the THEME program.

(A) VVT.VVT file	(B) .RDT file (Session 5)		(C) .RDT file (Session 29)	
HUM	Time	Event	Time	Event
R	5	:	5	:
EO	10	T,QA	10	T,MNT
DIS	15	M,RA	15	L,QA
ED	20	T,RP	20	G,RB
PD	25	R	25	T,MNT
NOI	30	M,EC	30	G,EO
MO	35	T,FF	35	R
S1	40	M,EC	40	D,CFR
S2	45	T,RP	45	G,EO
S3	50	M,EC	50	M,QA
TO	55	G,EC	55	T,MNT
TO	60	R	60	&
EX	65	&		
EE				
WHI				
S5				
ORD				
DO				
DIN				
QA				
QACL				
FF				
FO				
RP				
PV				
RT				
MNT				
MNT				
CFR				
CFR				
EXP				
RA				
EC				
CD				
RB				
DEF				
RD_N_P				
CT				
Q				
Q				
TURN				
O				
G				
D				
JM				
F				
L				
M				

****(A)****VVT.VVT file corresponding to the observation instrument*, ***(B)****.RDT file corresponding to session 5 (vignette from Table 2), and****(C)****.RDT file corresponding to session 29 (vignette from Table 3)*.*

**FIGURE 1:**
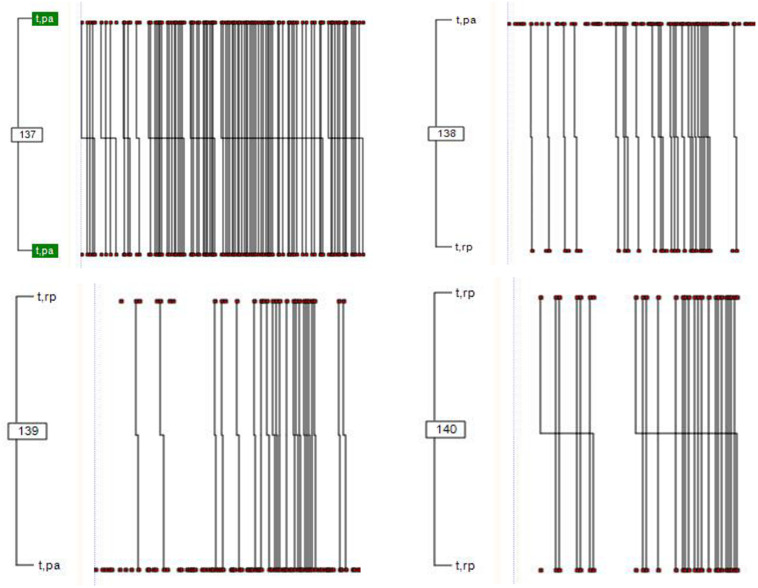
T-patterns in the initial session: minimum occurrence 30 and *p* < 0.005. Four T-patterns were identified linking turn-taking by the therapist to the questioning (QA, depicted as PA in the graph) and repetition/paraphrasing (RP) categories (both from the “facilitating conversation” dimension). The vertical lines correspond to each co-occurrence of *t* and *pa* behaviors (patterns 137 and 138), followed by co-occurrences of *t* and *pa* (pattern 137) and *t* and *rp* (pattern 138). The vertical lines also show co-occurrences of *t* and *rp* (patterns 139 and 140) followed *t* and *pa* (pattern 139), and *t* and *rp* (pattern 140). The length of the horizontal line fragments is proportional to their duration.

For the initial sesión (see Figure 1), we detected four T-patterns for the therapist as focal subject. These were related to two communication modalities: questioning (code QA) and repetition or paraphrasing (code RP).

**FIGURE 2:**
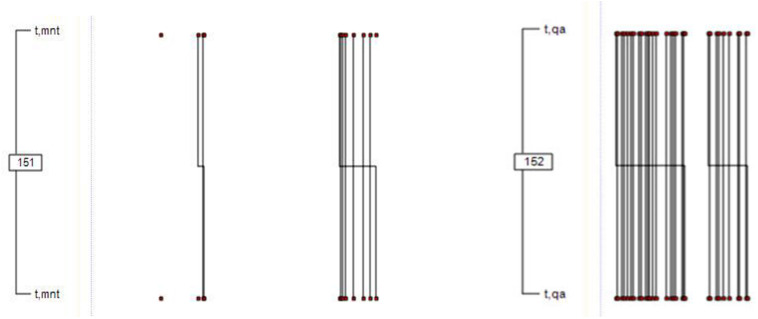
T-patterns in the final session: minimum occurrence 30 and *p* < 0.005. Two T-patterns were identified. One was linked to the turn-taking of the therapist in the mentalization (MNT) category (from the “mentalization” dimension), and the other was linked to the questioning (QA) category (from the “facilitating conversation” dimension). Vertical lines reflect co-occurrences of *t* and *mnt* (pattern 151) and *t* and *qa* (pattern 152) behaviors, followed by co-occurrences of *t* and *mnt* (pattern 151), and *t* and *qa* (pattern 152) behaviors. The length of the horizontal line fragments is proportional to their duration.

For the final sesión (see Figure 2), we detected two T-patterns, again related to two communication modalities: questioning (QA) and mentalization (MNT).

### Lag Sequential Analysis

For both sessions, sequential lag analysis was performed considering T as the criterion behavior and all other observation instrument codes as conditional behaviors. Table 6 shows the adjusted residual values obtained using the GSEQ5 program.

**TABLE 6:** Lag sequential analysis for the initial and final sessions, 5 and 29, respectively.

Session	Lag −3	Lag −2	Lag −1	Lag 0	Lag +1	Lag +2
Initial (5)	RP (2.13)	QA (4.52)	RP (9.22) MNT (3.34)	–	QA (4.78)	RP (4.24)
Final (29)	MNT (3.45)		RP (3.52)	MNT (8.52)	QA (2.83)	

**T (therapist) is the given behavior. RP indicates paraphrasing, QA, questioning, and MNT, mentalization. Numbers in parenthesis mean adjusted residual values (see Lag sequential analysis subsection)*.*

Sequential lag analysis of the data from session 5 revealed a behavioral pattern in which paraphrasing and use of questioning alternated between lags −3 and +2. A sequential pattern with mentalization located in the center (lag 0) was detected for session 29.

### Polar Coordinate Analysis

Obtained, considering T as the focal behavior and all other observation instrument codes as conditional behaviors, were parameters corresponding to the prospective and retrospective *Z*_*sum*_ values, from which vector length and angle values were calculated along with the quadrant in which the values were located. All values were obtained using HOISAN.

Tables 7, 8 show the parameters corresponding to sessions 5 and 29, respectively.

**TABLE 7:** Parameters corresponding to the prospective and retrospective *Z*_*sum*_ values obtained in session 5, considering T (therapist) as the focal behavior, from which vector length, vector angle, and quadrant were calculated.

Code	Quadrant	Prospective *Z*_*sum*_	Retrospective *Z*_*sum*_	Length vector	Angle vector
QA (facilitating conversation)	I	2.28	0.44	2.33(*)	10.97
RP (facilitating conversation)	I	2.69	4.23	5.01(*)	57.51
MNT (mentalization)	III	−1.76	−0.45	1.81	194.25

* means that vector is significative (>1,96).

**TABLE 8:** Parameters corresponding to the prospective and retrospective *Z*_*sum*_ values obtained in session 29, considering T (therapist) as the focal behavior, from which vector length, vector angle, and quadrant were calculated.

Code	Quadrant	Prospective *Z*_*sum*_	Retrospective *Z*_*sum*_	Length vector	Angle vector
QA (facilitating conversation)	I	5.23	2.85	5.95(*)	28.56
RP (facilitating conversation)	IV	0.4	−0.26	0.48	326.74
MNT (mentalization)	III	4.38	6.13	7.54(*)	54.45

* means that vector is significative (>1,96).

**FIGURE 3:**
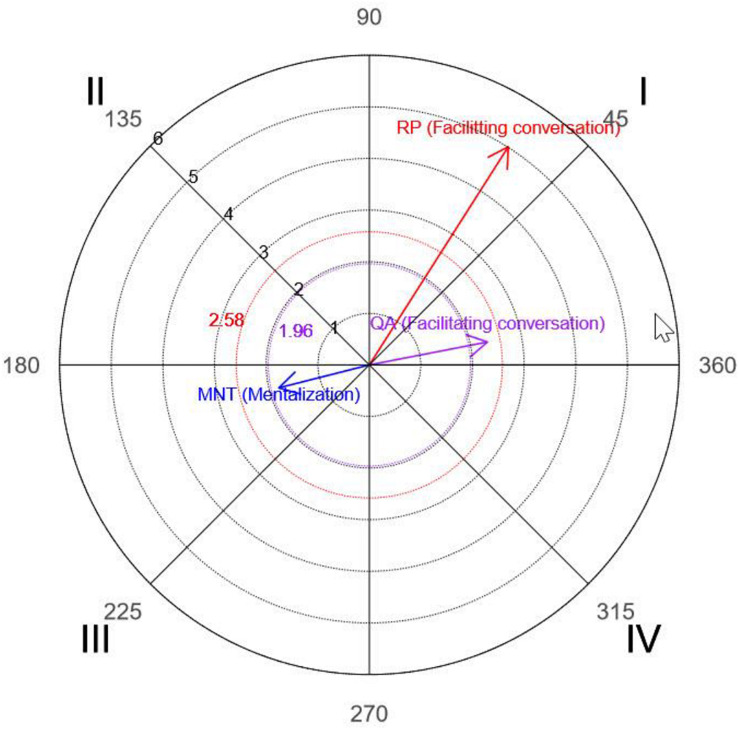
Polar coordinates for the initial session. The vectors correspond to interventions by the therapist (T) as focal behaviors and the communication strategies questioning (QA), paraphrasing (RP), and mentalization (MNT) as conditional behaviors.

**FIGURE 4:**
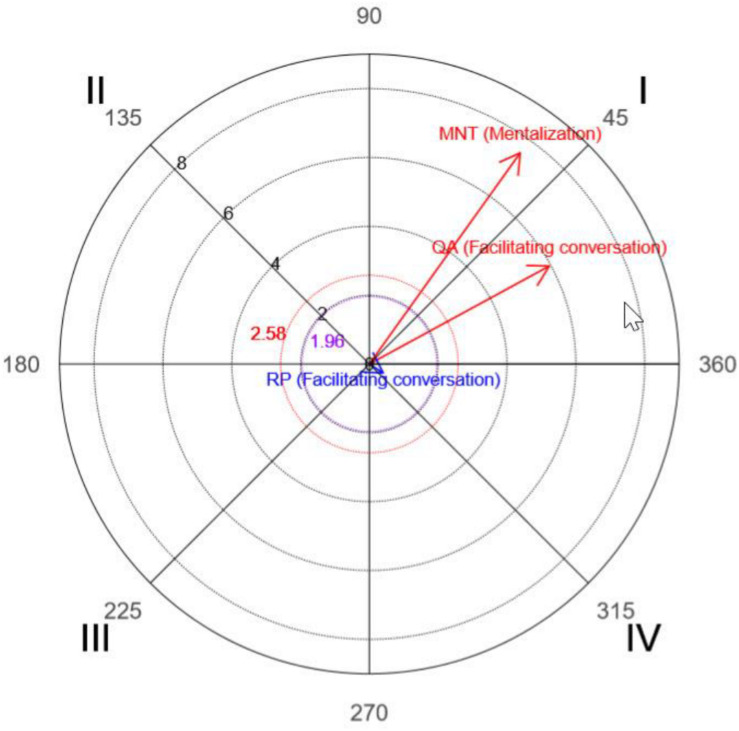
Polar coordinates for the final session. The vectors correspond to interventions by the therapist (T) as focal behaviors and the communication modalities questioning (QA), paraphrasing (RP), and mentalization (MNT) as conditional behaviors.

Graphs of the vectors, created using R, are depicted in Figures 3, 4.

The polar coordinate analysis showed that the therapist activated questioning and paraphrasing (quadrant I) in the initial session, and questioning and mentalization in the final session (quadrant I).

## Discussion

The results obtained in the T-pattern, lag sequential, and polar coordinate, analyses all show changes in the conversation-facilitation techniques used by the lead therapist from the start to the end of therapy. Although the combination of these three techniques has been used in different fields (e.g., Santoyo et al., 2017; Tarragó et al., 2017), this is the first time they have been applied in combination to clinical psychology. Our findings show that this is a remarkably productive approach to identifying relationships between communication modalities and changes that occur during the therapeutic process.

From the observation instrument composed of 15 dimensions and 28 categories (Table 1), three communication modalities in particular were identified – questions (QA), paraphrasing (RP), and mentalization (MNT) – suggesting that these are all powerful communication strategies for encouraging patient interaction in group therapy (Oetzel and Scherer, 2003).

Questioning by the therapist was observed in both the earlier and later sessions and its use shows that the therapist expressed interest in what the participants had to say, strengthening the therapist-patient bond. Questioning stimulates dialogue and encourages more inhibited group members to take the floor in a conversation and to express their experiences and feelings.

Paraphrasing was a particularly common strategy used in the initial session. Repeating what someone has said is a common technique for facilitating communication; it shows active listening and interest on the part of the therapist and facilitates an atmosphere of empathy and acceptance. This result corroborates the importance attached to reciprocity by adolescents as reported by Lavik et al. (2018). By repeating what the adolescent has just said, the therapist gives them the chance to continue talking ad treats them as an equal. In a sense, it constitutes a verbal reflection or “mirroring” of the speaker’s expressiveness that serves to hold attention and stimulate. Our findings, however, show that the therapist did not use this technique frequently, as it accounted for just 10% of her interventions, compared with 25% for questioning. The remaining 65% of interventions comprised a highly variable presence of the other 14 categories. Questioning and paraphrasing, both common strategies in psychotherapy, form part of the “facilitating conversation” dimension of the study observation instrument (Table 1).

Mentalization appeared in the final session, reflecting the communicative maturity of the group. In a previous study by our group, we found that paraphrasing was used to activate conversation from the early stages of therapy and also that it encouraged the emergence of mentalization (Arias-Pujol and Anguera, 2017). The results of the present study support this regulatory effect of paraphrasing as a prior requirement for the mentalization process (Fonagy et al., 2002); demonstrated is its relationship with the reflection or mirroring effect, while affect regulation and mentalization are linked to the development of self (Fonagy et al., 2018). In terms of the distinction between empathy with patients and challenging of their judgments by therapists, as described by Voutilainen et al. (2018), we saw that interventions designed to stimulate mentalization posed a true challenge to the adolescents in our group, who were found to “defend themselves” from this process, resorting to jokes and noises, touching, playful or more forceful hitting, laughter, and changes of subject. The therapist attempted to contain these emotions by non-judgmental interventions and by encouraging the adolescents to express themselves. The results suggest that a certain level of empathy and acceptance is necessary in psychotherapy to create an environment in which the therapist’s challenges are heeded and contribute to personal growth (Karver et al., 2006; Binder et al., 2011; Sagen et al., 2013).

From the clinical perspective, our results provide objective evidence, supported by three different analytical methods, of the important use that a therapist makes of three of the 28 communication strategies in the observation instrument, namely, questioning, repetition/paraphrasing, and interventions to improve mentalization. Unlike our previous study, which focused on differences in turn-taking in group sessions, this study focuses on differences in early and late communication strategies of the therapist. Our findings show how use of the different communication strategies varies from early to late therapy stages. In terms of novel findings, all three analytical methods detected a statistically significant increase in the use of questioning and repetition/paraphrasing as “conversation facilitators” in the early stages of therapy. In previous study (Arias-Pujol and Anguera, 2017), these strategies were grouped into a single block — conversation-facilitating DYN categories — formed by seven codes (FF, FO, RP, RT, QA, QC, and QV). A second novel finding, detected again by all three methods, was that the therapist made significantly greater use of questioning and mentalization in the later session compared to the earlier session to achieve the goals of the intervention.

This mixed methods study employed systematic observation and a succession of QUAL-QUAN-QUAL stages. We have shown that the combined use of T-pattern detection, polar coordinate analysis, and lag sequential analysis can offer meaningful and objective insights into what occurs in group psychotherapy from the angle of the therapist.

This study has three novel methodological aspects. First, it is the first to apply T-pattern detection to group psychotherapy with adolescents; second, it is first to combine T-pattern detection, polar coordinate analysis, and lag sequential analysis to analyze what occurs during the course of group psychotherapy from the perspective of the therapist; and finally, within the framework of a mixed methods study, our research incorporates one of the most powerful methods for linking qualitative and quantitative data, namely, the connecting method, which involves the systematized transformation of qualitative data into robust quantitative data for objective analysis.

## Data Availability Statement

All datasets generated for this study are included in the article.

## Ethics Statement

The studies involving human participants were reviewed and approved by the Head of the Eulàlia Torras de Beà Foundation (FETB) Research Department. Approval by an ethics committee was not required as per applicable institutional and national guidelines and regulations. Written informed consent to participate in this study was provided by the participants’ legal guardian/next of kin.

## Author Contributions

EA-P developed the project. MA conducted the method section and T-patterns, polar coordinate, and sequential analysis. Both authors participated in writing the article.

## Conflict of Interest

The authors declare that the research was conducted in the absence of any commercial or financial relationships that could be construed as a potential conflict of interest.

## Footnotes

1 https://www.mangold-international.com/en/products/software/gseq

2 www.patternvision.com

3 www.menpas.com
